# RETRACTION: Effects of Negative Pressure Wound Therapy on Wound Infection and Healing in Patients with Open Fracture Wounds: A Meta‐Analysis

**DOI:** 10.1111/iwj.70453

**Published:** 2025-03-26

## Abstract

**RETRACTION:** XuN.‐L. and MiW. , “Effects of Negative Pressure Wound Therapy on Wound Infection and Healing in Patients with Open Fracture Wounds: A Meta‐Analysis,” International Wound Journal 21, no. 2 (2024): e14757, 10.1111/iwj.14757.PMC1085060842052983

The above article, published online on 07 February 2024, in Wiley Online Library (http://onlinelibrary.wiley.com/), has been retracted by agreement between the journal Editor in Chief, Professor Keith Harding; and John Wiley & Sons Ltd. Following an investigation by the publisher, all parties have concluded that this article was accepted solely on the basis of a compromised peer review process. The editors have therefore decided to retract the article. The authors did not respond to our notice regarding the retraction.

**RETRACTION:** N.‐L. Xu and W. Mi , “Effects of Negative Pressure Wound Therapy on Wound Infection and Healing in Patients with Open Fracture Wounds: A Meta‐Analysis,” International Wound Journal 21, no. 2 (2024): e14757, 10.1111/iwj.14757.PMC1085060842052983

The above article, published online on 07 February 2024, in Wiley Online Library (http://onlinelibrary.wiley.com/), has been retracted by agreement between the journal Editor in Chief, Professor Keith Harding; and John Wiley & Sons Ltd. Following an investigation by the publisher, all parties have concluded that this article was accepted solely on the basis of a compromised peer review process. The editors have therefore decided to retract the article. The authors did not respond to our notice regarding the retraction.

